# Depressive Symptomatology and Practice of Safety Measures among Undergraduate Students during COVID-19: Impact of Gender

**DOI:** 10.3390/ijerph18094924

**Published:** 2021-05-05

**Authors:** Badr K. Aldhmadi, Rakesh Kumar, Ramaiah Itumalla, Bilesha Perera

**Affiliations:** Department of Health Management, College of Public Health and Health Informatics, University of Ha’il, Ha’il 81451, Saudi Arabia; ra.kumar@uoh.edu.sa (R.K.); r.itumalla@uoh.edu.sa (R.I.); bileshap@gmail.com (B.P.)

**Keywords:** depressive symptoms, university undergraduates, Saudi Arabia, precautionary measures, COVID-19

## Abstract

The COVID-19 pandemic has greatly affected the personal and academic lives of undergraduates in Saudi Arabia. Although studies have suggested that COVID-19 increased the prevalence of psychological health problems among undergraduates, the associations between the risk of depression and safety practices and the influence of gender on these associations have not been studied in detail. A cross-sectional online survey was conducted among preparatory-year undergraduates in a large public university in Saudi Arabia during the outbreak. Depressive symptoms were assessed using the Center for Epidemiological Studies Depression (CES-D) Scale, and the practice of eight precautionary behaviors was also assessed. Data analysis was performed using the chi-square test, multiple linear regression and Spearman’s correlation coefficient. In total, 3044 undergraduates were surveyed. The mean age was 18.6 years (*SD* = 0.84), and 61.9% (*n* = 1883) of the participants were female. Overall, 47.7% of the participants reported having elevated depressive symptoms. Overall mean values of CES-D scores were higher among female undergraduates than that of male undergraduates (18.08 versus 15.56, *p* < 0.01). There were inverse and weak but significant relationships between the CES-D score and frequent cleaning of hands (male: *r* = −0.116, *p* < 0.01; female: *r* = −0.098, *p* < 0.01), wearing a mask when going out (male: *r* = −0.172, *p* < 0.01; female: *r* = −0.135, *p* < 0.01), keeping social distance (male: *r* = −0.117, *p* < 0.01; female: *r* = −0.147, *p* < 0.01), and covering the nose when sneezing (male: *r* = −0.202, *p* < 0.01; female: *r* = −0.115, *p* < 0.01). Regression analysis indicated that adherence to precautionary measures was a strong predictor of reduction of depressive symptoms in the target population. Male gender was also found to be an independent predictor of reduction of depressive symptoms. Depressive symptoms were highly prevalent in this target group, and female undergraduates seemed to be more vulnerable to developing such symptoms. Results also indicated that female undergraduates were more likely to implement the protective measures for COVID-19. The promotion of precautionary measures seems to be effective in reducing distress in this target population, but further research is needed to confirm our assertions.

## 1. Introduction

The COVID-19 disease, which is caused by the severe acute respiratory syndrome coronavirus 2 (SARS-CoV-2), has already had devastating health and socio-economic consequences on the world population [1,2,3]. The first COVID-19 case was detected in Wuhan, China in December 2019, and at present, people in almost all countries in the world are affected by the disease. By the end of 2020, 1.8 million deaths were recorded worldwide due to COVID-19 [4]. A number of strategies were implemented by countries across the world to mitigate the spread of the disease. Lockdown of cities or the whole country, imposing curfew, banning social and religious gatherings, closure of educational institutions, and public education about personal safety measures against the disease such as hand hygiene and wearing masks were some of the strategies implemented by countries [1,5,6].

The first case of COVID-19 was detected in Saudi Arabia on 2 March 2020. City-wide lockdowns, curfews, school and university closures, transportation restrictions, community screening, and the promotion of the adoption of safety measures were some of the preventive strategies implemented by the Saudi government to control the spread of the COVID-19 [6,7]. Within a week of the first case of COVID-19 being found, schools, universities, and other educational institutions were closed to prevent the spread of COVID-19 across the country [7,8]. As per the directions of the Minister of Education, virtual schools and distance education were initiated nationwide [8], and distance learning methods were quickly implemented to run the university education in Saudi Arabia without disturbances. These actions might have caused uneasiness and distress in the energetic and socially and physically active population group of university undergraduates, and brought about a new set of challenges for the group [9,10]. The higher number of assignments that the students had to complete, failures in understanding the requirements of some of those assignments and subjectivity of some of those marking processes, internet connection problems, unavailability of study materials and lab facilities, and limited student–teacher interactions were found to be major distressing factors associated with the shift from learning on campus to distance learning. Many such factors have created anxieties and fear of delays in graduating and subsequent unemployment and financial insecurity issues among undergraduates. Furthermore, social distancing, limited social gatherings, quarantine measures, and mandatory face coverings have caused undergraduate lifestyles and work plans to be disturbed, causing elevated levels of stress and fear among undergraduates. A number of studies have already examined the indirect impact of the COVID-19 outbreak on university undergraduates’ academic and psychosocial functions. Anxiety, depressive symptoms, suicidal ideation, sleeping difficulties, and concentration difficulties were found to be common among these students during the pandemic [11,12,13]. Studies have also found that adherence to stay-at-home orders and feeling socially connected during the COVID-19 lockdown acted as protective factors against poor psychological health among undergraduates [13,14].

Gender differences in social relations, educational and work opportunities have been observed in Saudi Arabia [15]. Societal norms limit females’ outdoor exercise and social gatherings and Saudi Arabian law requires a male relative’s agreement before seeking work, education, travel or issuing an identity card or passport. Although about 50% of the Saudi population is female, it is observed that barely 21% of them contribute to social development, because it is socially unacceptable for women to work in some fields. These circumstances may have a negative impact on the health and well-being of women in Saudi Arabia. Thus, it is imperative to investigate gender perspectives in relation to the development of depressive symptoms and precautionary behavioral acts among university undergraduates in Saudi Arabia. Such information would greatly assist public health authorities in the country to develop effective strategies to curb this menace.

It has been observed that the mortality rates of COVID-19 in men were higher than those of women [16,17]. Factors including biological attributes, such as immune system responses, and behavioral factors, such as smoking, occupation, and healthcare-seeking behaviors, have been suggested as possible causes of such a gender difference in COVID-19 mortality and risk of infection [16,18]. It has been observed that women are superior to men in dealing positively with infectious diseases [19]. Although gender plays a vital role in those infected with COVID-19, this important demographic factor has not been evaluated thoroughly with respect to acquiring COVID-19 infection in adolescents and young adults. Furthermore, it has been observed that there is an unmet need for psychological support for university undergraduates [11,12,20]. Thus, research on gender differences of COVID-19 psychological consequences and adherence to precautionary measures is urgently needed to understand the current scenario and to remedy the adverse consequences. In this study, depressive symptomatology and behavioral protective actions against COVID-19 undertaken by first-year university undergraduates in one of the largest public universities in Saudi Arabia and the role played by gender in the relationships between these variables were examined.

## 2. Methods

A web-based online cross-sectional survey was conducted between 15 August and 15 September 2020, on a sample of first-year university undergraduates at one of Saudi Arabia’s largest public universities, in Ha’il. An anonymous Google form was used. The survey questionnaire assessed the following demographic characteristics: age, gender, year of study, number of family members, and monthly family income. Depressive symptoms were assessed using the Center for Epidemiological Studies Depression (CES-D) Scale, which has been developed to screen for depressive symptoms in community settings [21]. The CES-D is a reliable and valid scale that has been widely used in surveys conducted among adolescents and young adults [22]. The tool is focused on the experiences of the respondent in the past week and has a four-factor and 20-item structure. Depressive symptoms are assessed through self-statements such as “I felt hopeful about the future”. Total scores range from 0 to 60, and the cutoff score is 16. Those with scores ≥16 are considered to have an elevated level of depressive symptoms. Furthermore, those who scored 16–23 and those who scored ≥24 were classified as “moderate” and “severe” cases of depressive symptomatology, respectively. The practice of nine precautionary behaviors was assessed using a 1 to 5 scale (1 = yes, almost always to 5 = no, not at all). The eight precautionary measures are use of sanitizers, wearing a mask, limiting travel, staying at home most of the time, reading news related to COVID-19, avoiding common public places, keeping social distance, and covering the nose when sneezing.

The questionnaire was developed in English and subsequently translated into Arabic to ensure that accurate responses were elicited from the participants. During the questionnaire development process, the items were revised and amended by conducting a pilot survey and soliciting expert feedback. This study was approved by the Ethics Review Committee of the University of Ha’il, Saudi Arabia (Ethics Review Number: H-2020-093). The study population was 1st year undergraduates in Ha’il region in Saudi Arabia. Data were collected anonymously and all data that were collected were stored electronically in the personal computer of the principal author. Only co-investigators were allowed to examine the data set. Participants were informed that participation is completely voluntary. Random sampling techniques could not be used because classroom teaching had been suspended in Saudi Arabia at the time of data collection. Therefore, an electronic platform was used, and the questionnaire was sent to students electronically. Snowball sampling was used to recruit as many respondents as possible. Through our professional and personal networks, we contacted class representatives and requested them to forward the survey link to the undergraduate students of the University of Ha’il through WhatsApp messenger. Internet Protocol address (IP) of the respondent computer was used to identify potential duplicate entries from the same user [23]. Statistical Package for Social Sciences (SPSS) version 25 (IBM Corp, Armonk, NY, USA) was used to conduct statistical analyses. The level of statistical significance was set as *p* < 0.05. Frequencies and percentages were computed to examine categorical variables, and means and standard deviations were computed to examine continuous variables. Spearman’s correlation coefficients were computed to examine the relationships between ordered categorical and continuous variables. The Cronbach’s alpha of the nine items that assessed the practices of precautionary behaviors was 0.76, indicating an acceptable internal consistency.

## 3. Results

A total of 3044 undergraduates were surveyed. The mean age was 18.6 years (*SD* = 0.84), and 61.9% (*n* = 1883) of them were female. About 72% of the participants reported having monthly family income of less than or equal to SR 20,000, 22% between SR 20,001 to SR 50,000 and the remaining 6% more than SR 50,000. About 25% of the participants had a family size of six or fewer members, while 60% had 7–10 members in their families, and the rest (15%) had more than 10 members in their families.

The participants’ actual precautionary behaviors with respect to the pandemic are given in Table 1. About four-fifths of both male and female undergraduates adhered to wearing a mask when going out and covering the nose when sneezing. Only about two-thirds of both male and female participants reported that they often use hand sanitizers and maintain social distancing when going out. These four precautionary measures were the most important and effective behavioral actions promoted by the Saudi government in controlling the spread of the disease. A slightly higher percentage of male undergraduates compared to female undergraduates reported wearing masks when going out (83.7% versus 74.1%, *p* < 0.01). Female undergraduates were more likely than male undergraduates to limit travelling (59.6% versus 51.1%, *p* < 0.01) and stay at home (75.4% versus 62.0%, *p* < 0.01). Notably, only about one-quarter of both male and female undergraduates reported acquiring information regarding COVID-19 from printed and electronic media.

Overall, 47.7% of the participants were found to have elevated (moderate and severe) depressive symptoms. Furthermore, it was observed that male undergraduates were more likely to develop elevated depressive symptoms than female undergraduates (Table 2). Among males, 27.1% were found to have severe depressive symptoms and among females the corresponding figure was 20.7% (*p* < 0.05). However, the overall mean values of CES-D scores were higher among female undergraduates than that of male undergraduates (18.08 versus 15.56, *p* < 0.01).

The government of Saudi Arabia has implemented rigorous public health measures to mitigate the spread of COVID-19 in the Kingdom. Among them, frequent cleaning of hands, wearing a mask, keeping social distance, and covering the nose when sneezing were the leading behavioral protective actions promulgated. There were inverse and weak but significant relationships between CES-D score and significant frequent cleaning of hands (male: = −0.116, *p* < 0.01; female: *r* = −0.098, *p* < 0.01), wearing a mask when going out (male: *r* = −0.172, *p* < 0.01; female: *r* = −0.135, *p* < 0.01), keeping social distance (male: *r* = −0.117, *p* < 0.01; female: *r* = −0.147, *p* < 0.01), and covering the nose when sneezing (male: *r* = −0.202, *p* < 0.01; female: *r* = −0.115, *p* < 0.01) (Table 3 and Table 4).

Stepwise multiple linear regression technique was applied to examine the predictors of depressive symptoms. Precautionary behavioral acts, gender, family income and number of family members were the predicting variables investigated. A total score for precautionary behavioral acts was calculated by adding the number allocated to the answers given. Thus, higher scores in this total score indicate less obedience to precautionary measures. In multiple linear regression, precautionary measures, gender, family income and number of family members were added to the model stepwise to identify independent and confounding variables (Table 5). 

It was observed that precautionary behavioral acts and gender were strong independent predictors of CES-D scores.

## 4. Discussion

Assessment of young university undergraduates’ actual behavior with respect to the COVID-19 pandemic is important to design and implement protective measures against the pandemic in higher education institutions in Saudi Arabia. Gender has been identified as a vital factor that needs to be considered in any health promotion activity. COVID-19-related safety practices promoted by international health agencies have been criticized due to their cultural insensitivity and implementation issues in non-Western societies where gender plays a major role [24,25]. Although hand hygiene practices and covering the nose when sneezing were prevalent behaviors in Western societies even before the pandemic, such practices were not as common in non-Western societies, especially among people in poor socio-economic brackets. This would probably explain why nearly one-third of university undergraduates did not adhere to hand hygiene practices during the COVID-19 period in Saudi Arabia. In Saudi Arabia, women often cover their entire face except eyes when they go out. Wearing of a special face veil called Abaya by women has been in practice in Islamic societies for centuries. Thus, wearing a mask may not be practical for them and the majority may have believed that an Abaya would serve the mask’s purpose. This may explain why nearly one-quarter of female undergraduates did not comply with this behavioral action. A higher percentage of male participants (84%) reported compliance with this behavioral action. In Saudi Arabia men, compared to women, are highly mobile. A higher percentage of female undergraduates, therefore, tend to have fewer interactions with outside people, as was observed in this research. Only about 64% of the respondents reported practicing social distancing, and no gender difference was found. It is important for the government to take necessary steps to implement this public health action in all common places, which was proven to be highly effective in controlling the pandemic. About four-fifths of both male and female undergraduates reported that they cover their noses when sneezing, another vital precautionary behavior promoted by the government. Overall, male undergraduates were less likely to report protective actions against COVID-19, as observed in some other studies [24,26]. Young male undergraduates’ cultural and religious norms and beliefs, as well as their active lifestyles, may have influenced them in not adhering to personal protective measures against COVID-19 that seem to restrict their freedom.

Given that nearly half of the participants in this study had elevated depressive symptoms, where the figure was higher than in normal circumstances, universities should pay more attention to identifying and providing necessary support for undergraduates with psychological problems during a pandemic. It has been found that the demand for psychological health services in universities was high during the pandemic period and the relevance of digital mental healthcare during COVID-19 has been highlighted [27,28,29]. University undergraduates tend to exhibit higher rates of depressive symptoms compared to non-undergraduate peers during the pandemic [29,30,31]. This study’s results also indicate a considerable gender difference in the prevalence of depressive symptoms among the participants. Many studies have indicated that women are at higher risk of developing depressive symptoms [29,30], and the same was observed among female undergraduates during the pandemic [29]. Although, a slightly higher percentage of male undergraduates reported elevated depressive symptoms than that of female undergraduates in our study sample, the mean score of the CES-D is significantly higher in female undergraduates than that of male undergraduates. The socio-cultural underpinnings of Saudi people may explain this. Women in Saudi Arabia have greater restrictions on their movement than men. This may have resulted in women having less opportunity to interact with outside people and to mitigate their stress and fear of contacting COVID-19. As a result, female undergraduates may make extra effort to practice precautionary behaviors. Nevertheless, sudden lifestyle changes, disruption of academic and social activities, and uncertainties about the future have had a negative impact on the psychological health of most university undergraduates irrespective of gender [9,11,31,32]. However, gender-specific interventions which are culturally sensitive should be promoted to mitigate the risk of COVID-19 and developing stresses related to the disease. Online counseling services and mental health promotion programs are needed for vulnerable students during sudden disease outbreaks, and higher education authorities should pay urgent attention to this issue, which is associated with academic performance and the well-being of university undergraduates.

As expected, in both male and female participants, a negative relationship was observed between depressive symptomatology and adherence to safety measures. This is, however, not in line with the observations made in some similar studies, where participants with high adherence to precautionary measures were found to exhibit higher rates of depressive symptoms [26,29], but it is in line with some other studies where higher compliance was found to be related to lower depression [33]. One possible reason for our study observation is that students’ confidence about government-implemented precautionary measures may have lowered their stress levels in relation to the disease. Nevertheless, it is not clear whether higher adherence to precautionary measures leads to a lower level of depressive symptoms, or whether elevated depressive symptoms decrease adherence. It is vital to explore these research gaps related to COVID-19, and implement and fund research agendas which are novel and impactful [30]. Findings of this research would serve this purpose. Further research, however, is warranted to elucidate underlying causal mechanisms in this target population.

There were several limitations to this study. The participants were selected through non-random sampling due to time constraints and COVID-19-related restrictions imposed in the university. The use of an online questionnaire may have caused the participants to respond with socially desirable responses. Nevertheless, the study used a standardized scale to assess depressive symptoms and the analysis was based on a very large sample, which can provide more accurate mean values and a smaller margin of error. Furthermore, the time period of data collection was highly appropriate for the study’s objectives.

## 5. Conclusions

The adherence to precautionary measures during the COVID-19 pandemic by Saudi university undergraduates is satisfactory. The findings indicate that female undergraduates were more likely to implement the protective measures than their male counterparts. Our results further indicate that adherence to precautionary measures may help protect undergraduates from becoming depressed, but further research is needed to confirm this assertion. Targeted psychological health promotion interventions that take gender into consideration are urgently needed during the fight against COVID-19 among university undergraduates, to mitigate psychological distresses that may adversely affect their academic performance. 

## Figures and Tables

**Table 1:** Percentage of undergraduates who adhered to the safety measures of COVID-19 by gender (*n* = 3044).

Practice of Safety Measures	Male	Female	Chi-Square Value	*p* Value
I wash my hands or use sanitizers often	66.2%	66.1%	0.005	*p* > 0.05
I wear a mask when I go out	83.7%	74.1%	39.05	*p* < 0.01
I have limited my travelling	51.1%	59.6%	21.25	*p* < 0.01
I stay at home most of the time	62.0%	75.4%	62.57	*p* < 0.01
I read/watch news related to coronavirus	27.2%	25.7%	0.79	*p* > 0.05
I avoid common places like supermarkets	41.4%	44.9%	3.70	*p* < 0.05
I try to keep at least 3 feet away from people when I go out	63.2%	65.9%	2.35	*p* > 0.05
I cover my nose when I sneeze	81.8%	86.5%	12.24	*p* < 0.01

**Table 2:** Prevalence of depressive symptoms by gender.

Depressive Symptoms	Male (*n* = 1161) Percentage (%) 95% CI	Female (*n* = 1883) Percentage (%) 95% CI
No or mild depressive symptoms	48.3 (48.27, 48.32)	58.8 (58.77, 58.82)
Moderate depressive symptoms	24.6 (24.58, 24.61)	20.5 (20.47, 20.52)
Severe depressive symptoms	27.1 (27.07, 27.12)	20.7 (20.67, 20.72)

**Table 3:** Correlations between depressive symptoms and adherence to precautionary measures among male undergraduates (*n* = 1161).

	CES-D	I Wash My Hands or Use Sanitizers Often	I Wear a Mask When I Go Out	I Try to Keep at Least 3 Feet away from People When I Go Out	I Cover My Nose When I Sneeze
CES-D	1.000				
I wash my hands or use sanitizers often	−0.116 **	1.000			
I wear a mask when I go out	−0.172 **	0.521**	1.000		
I try to keep at least 3 feet away from people when I go out	−0.117 **	0.457 **	0.384 **	1.000	.
I cover my nose when sneezing	−0.202 **	0.308 **	0.341 **	0.380 **	1.000

** *p* < 0.01.

**Table 4:** Correlations between depressive symptoms and adherence to precautionary measures among female undergraduates (*n* = 1883).

	CES-D	I Wash My Hands or Use Sanitizers Often	I Wear a Mask When I Go Out	I Try to Keep at Least 3 Feet away from People When I Go Out	I Cover My Nose When I Sneeze
CES-D	1.000				
I wash my hands or use sanitizers often	−0.098 **	1.000			
I wear a mask when I go out	−0.135 **	0.502 **	1.000		
I try to keep at least 3 feet away from people when I go out	−0.147 **	0.419 **	0.476 **	1.000	
I cover my nose when sneezing	−0.115 **	0.285 **	0.348 **	0.392 **	1.000

** *p* < 0.01.

**Table 5:** Regression coefficients for predicting depressive symptoms.

Variable	B	95% CI for B	β	t	*p*
Precautionary Behavioral acts	0.143	(0.078–0.207)	0.078	4.33	0.001
Gender	2.48	(1.672–3.296)	0.109	5.99	0.001
Income	0.571	(−0.106–1.248)	0.030	1.65	0.098
Number of family members	−0.144	(−0.296–0.008)	−0.033	−1.86	0.063

## Data Availability

The data presented in this study are available on reasonable request from the corresponding author.
